# M2e-Derived Peptidyl and Peptide Amphiphile Micelles as Novel Influenza Vaccines

**DOI:** 10.3390/ph17111503

**Published:** 2024-11-08

**Authors:** Megan C. Schulte, Agustin T. Barcellona, Xiaofei Wang, Adam G. Schrum, Bret D. Ulery

**Affiliations:** 1 Department of Chemical and Biomedical Engineering, University of Missouri, Columbia, MO 65211, USA; mcsc2d@umsystem.edu (M.C.S.); atbbtf@missouri.edu (A.T.B.); schruma@health.missouri.edu (A.G.S.); 2 Department of Molecular Microbiology and Immunology, University of Missouri, Columbia, MO 65211, USA; 3 Department of Surgery, University of Missouri, Columbia, MO 65211, USA; 4 NextGen Precision Health Institute, University of Missouri, Columbia, MO 65211, USA; 5 Materials Science & Engineering Institute, University of Missouri, Columbia, MO 65211, USA

**Keywords:** vaccine, micelle, influenza, M2, peptide amphiphile

## Abstract

**Background:** A significant problem with current influenza vaccines is their reliance on predictions of the most prevalent strains for the upcoming season, with inaccurate forecasts greatly reducing the overall efficacy of the immunization campaign. A universal influenza vaccine, which leverages epitopes conserved across many, if not all, strains of influenza, could reduce the need for extremely accurate forecasting. The highly conserved ectodomain of the influenza M2 protein contains a B cell epitope in the M2_2–16_ region, making it a promising candidate as a universal influenza vaccine. Unfortunately, free peptide antigens alone are limited as vaccines due to their poor stability and weak immunogenicity in vivo. To improve the potential of peptide vaccines, immunostimulatory micellar nanoparticles can be generated from them by lipid conjugation (i.e., peptide amphiphiles—PAs). **Methods:** M2_2–16_ peptides and Palm_2_K-M2_2–16_-(KE)_4_ PAs were synthesized and characterized. BALB/c mice were subcutaneously vaccinated with these formulations, and ELISAs were conducted on serum collected from the vaccinated mice to evaluate induced antibody responses. **Results:** Unlike other peptide antigens previously studied, the unmodified M2_2–16_ peptide micellized without any peptidyl or lipid modifications. M2_2–16_ peptidyl micelles (PMs) were spherical with largely undefined secondary structure somewhat different from the cylindrical, β-sheet-containing Palm_2_K-M2_2–16_-(KE)_4_ peptide amphiphile micelles (PAMs). Differences in physical properties were found to correlate with slightly different immune responses with PAMs eliciting higher antibody titers after the initial immunization, whereas both micelle types elicited strong IgG titers after a prime-boost regimen. **Conclusions:** These results suggest the viability of PAMs as single-dose vaccines, while both PMs and PAMs show potential using a multi-dose immunization approach.

## 1. Introduction

Despite existing treatments and vaccines, influenza remains an ongoing global problem. During the 2022–2023 influenza season, there were an estimated 26–51 million influenza-related illnesses and 18,000–97,000 deaths in the United States alone [1]. Two substantial weaknesses with our current influenza vaccine strategy employing inactivated virus are the long vaccine production times and, consequently, the need for forecasting the prevailing strains for the next influenza season. Vaccine efficacy suffers when, as a result of inaccurate predictions, the strains in the influenza vaccine do not match the dominant strains in a given influenza season [2,3,4,5]. To address these shortcomings, there have been extensive efforts in the development of a universal influenza vaccine that can protect against a broad spectrum of viral strains. This approach can be best achieved by harnessing conserved epitopes that are consistent across several strains of influenza. For example, the external region of the M2 ion channel protein (M2e, the first twenty-four amino acids of the protein) is one such region, with a high degree of similarity across several influenza strains including H1N1, H3N2, and H2N2 [6,7,8], all ones with high pandemic potential. Excitingly, this region, more specifically M2_2–16_, has been shown to contain a B cell epitope, making it an attractive candidate as a universal vaccine [9,10,11].

Subunit vaccines leverage individual antigenic components in the form of recombinant nucleic acids, recombinant proteins, or synthetic peptides (e.g., M2_2–16_) instead of using the entire pathogen, offering an appealing alternative to whole virus and split virus vaccine. Because the antigenic components are specifically chosen to include only the absolutely necessary immunogenic portions of the virus, there is no concern for hypersensitivities to unnecessary viral components, gelatin (a common viral stabilizer), or egg proteins, which, although rare, have been reported in egg-based vaccines [12,13]. Secondly, the production time of subunit vaccines can be significantly shorter than that of egg-based vaccines, which is especially relevant for rapidly mutating viruses like influenza [14]. Peptide vaccines in particular can be produced via solid-phase synthesis, which is much faster than growing whole virus vaccines in eggs or other vectors and does not directly compete with food or agricultural industries. Additionally, peptides, especially when lyophilized, have the benefit of remaining functional at room temperature for days to weeks and, when refrigerated, for years, with reconstitution being as simple as adding buffered solutions and mixing to solubilize them [15,16]. This stability has the potential to make peptide vaccines more accessible to underdeveloped communities with less reliable cold chain storage systems than nucleic acid or protein vaccines, which can denature in inconsistent or warmer temperatures [15,17]. However, peptide vaccines also have disadvantages, including lacking inherent immunogenicity, often being unable to maintain their native conformation, and being susceptible to protease-mediated degradation in the body [18,19].

Many of the disadvantages of peptide vaccines can be abrogated by packaging peptides into a delivery vehicle such as a nanoparticle. One of the easier approaches for incorporating peptides into a nanoparticle-based system is to induce peptide self-assembly, such as through micellization. Micelles are self-assembled structures that are held together by supramolecular forces including hydrophobic, ionic, and other weaker interactions. Compared to free peptides, products that form such ultrastructures are less susceptible to enzymatic degradation and have a more defined secondary structure [20,21,22]. Importantly, the multivalent display of antigens on the micelle surface and improved peptide-cell association increase the immunogenicity of the antigen [23,24,25,26,27]. Previous work has shown that micelle size and morphology dictate their immunogenicity and these characteristics can be tuned by a number of factors including the addition of lipids and the incorporation of hydrophilic residues [28]. Specifically, it has been shown that peptide amphiphiles (PAs) with dipalmitoyllysine on their N-terminus and a zwitterion-like charge block on their C-terminus (i.e., Palm_2_K-antigen-(KE)_4_) self-assemble into peptide amphiphile micelles (PAMs) that induce the highest IgG antibody titers in vivo [25]. PAM immunogenicity can be further improved through the incorporation of the TLR-2 agonist adjuvant Pam_2_CSK_4_ [25,29,30]. Altogether, Palm_2_K-antigen-(KE)_4_ micelles co-delivered with Pam_2_CSK_4_ have the potential to be ideal carriers to be leveraged as a universal influenza vaccine platform. Thus, this work sought to investigate the enhanced immunogenicity of the M2_2–16_ peptide antigen in the context of micellization and adjuvant incorporation.

## 2. Materials & Methods

### 2.1. Peptide and Peptide Amphiphile Synthesis and Purification

The TLR2 agonist Pam_2_CSK_4_ was purchased from Invivogen (San Diego, CA, USA). The peptides M2_2–16_, M2_2–16_-(KE)_4_, and M2e (where M2_2–16_ is SLLTEVETPIRNEWG, (KE)_4_ is KEKEKEKE, and M2e is the full protein ectodomain of MSLLTEVETPIRNEWGCRCNDSSD) were synthesized using Fmoc solid-phase peptide synthesis on Sieber Amide resin using a Tetras Peptide Synthesizer (Louisville, KY, USA). Nα-Fmoc-L-amino acids with acid-labile protecting groups on their reactive side chains were used to build the peptides studied in this research. Fmoc protecting groups were removed using 6% piperazine and 0.1 M hydroxybenzotriazole (HOBt) in dimethylformamide (DMF). Monomer couplings were done using at least 3 equivalents monomer, 6 equivalents N,N-diisopropylethylamine (DIPEA), 2.7 equivalents hexafluorophosphate benzotriazole tetramethyl uranium (HBTU), and 3 equivalents HOBt in N-methyl-2-pyrrolidone (NMP) compared to 1 molar equivalent of peptide-on-resin. After each coupling step, 5% acetic anhydride and 7% DIPEA in NMP was added to the resin to acetylate any remaining free amines. To generate PAs, a dipalmitoyllysine group (Palm_2_K) was added to the N-terminus of peptides by coupling palmitic acids to a peptide containing a non-native N-terminal deprotected Fmoc-Lysine(Fmoc). For fluorophore-labeling, 5(6)-carboxyfluorescein (FAM) or 5(6)-carboxytetramethylrhodamine (TAMRA) were attached either on the N-terminus of a non-lipidated peptide or, in the case of the PA, to the deprotected ε-amine of a Lysine(Dde) added to the bioactive peptide N-terminus before the inclusion of the dipalmitoyllysine group. Dde was removed with 2% hydrazine in DMF. Fluorophores were coupled to the peptide using the same protocol as amino acid addition.

Peptides were cleaved from resin using a cleavage cocktail of 2.5% each of water, phenol, triisopropylsilane, thioanisole, and ethane-1,2-dithiol in trifluoroacetic acid (TFA) for at least 2 h, then precipitated in ether. Peptides were purified on a reverse-phase C18 column (for non-lipidated peptides) or C4 column (for lipidated peptides) in a mobile phase of water, acetonitrile, and 0.1% TFA using a gradient of increasing acetonitrile content. M2_2–16_, M2e, and Palm_2_K-M2_2–16_-(KE)_4_ eluted at approximately 35%, 35%, and 65% acetonitrile, respectively. Fluorophore-labeled FAM-M2_2–16_, Palm_2_K-K(FAM)-M2_2–16_-(KE)_4_, and TAMRA-Pam_2_CSK_4_ products eluted at approximately 40%, 60%, and 60% acetonitrile, respectively. The purified fractions were lyophilized and analyzed using mass spectrometry-controlled high-performance liquid chromatography (LC-MS, Beckman Coulter (Brea, CA, USA)) to a purity of greater than 90% (Figure S1).

### 2.2. Critical Micelle Concentration

Critical micelle concentration (CMC) was indirectly determined by measuring the change in fluorescence that occurs when 1,6-diphenylhexatriene (DPH) becomes encapsulated in the core of micelles. A solution of 100 mM DPH in tetrahydrofuran (THF) was prepared and then diluted 100-fold in deionized, distilled water (ddH_2_O). The resulting solution was diluted 1000-fold in phosphate-buffered saline (PBS) to generate a 1 μM DPH solution which was used to serially dilute a 1 mM peptide or PA in ddH_2_O solution from 100 μM to 0.92 nM. After a one-hour incubation in the dark, fluorescence of the serial dilutions was measured using an excitation wavelength of 350 nm and an emission wavelength of 428 nm employing a Biotek Cytation 5 Spectrophotometer (Santa Clara, CA, USA). The CMC for a given product was determined to be the intersection of two logarithmic regression lines on a plot of fluorescence intensity versus peptide concentration. The first line (below the CMC) was fit to at least four points where the slope is relatively flat and the second line (above the CMC) was fit to at least three points which had a slope roughly ten times that of the first line, thus indicating a substantial change in fluorescence caused by DPH entrapment within the micelle core.

### 2.3. Transmission Electron Microscopy

Solutions of 20 μM peptide or PA with or without 2.22 μM Pam_2_CSK_4_ were prepared in PBS from which 5 μL was pipetted onto a carbon-coated copper transmission electron microscopy (TEM) grid and incubated for 3–5 min before excess solution was wicked away using filter paper. Then, 5 μL Nano-W (2% methylamine tungstate) was pipetted on the grid and incubated for 3–5 min before being removed by wicking. Grids were imaged using a JEOL JEM-1400 Transmission Electron Microscope (Tokyo, Japan) at 15,000× and 25,000× magnification. Micrographs of peptide or PA were compared against reference micrographs of Nano-W with or without PBS to ensure proper identification of micellar structures.

### 2.4. Circular Dichroism

The circular dichroisms (CDs) of peptide and PA solutions (250 μM) were measured from 190 nm to 250 nm with a step size of 0.1 nm on a Jasco J-1500 Circular Dichroism Spectrophotometer (Oklahoma City, OK, USA). The data were fit to reference CD curves of poly(lysine) and poly(glutamine) to approximate α-helix, β-sheet, and random coil content of the samples. Average percentage plus/minus standard deviation of each secondary structure over three to four runs is reported.

### 2.5. Förster Resonance Energy Transfer

Förster resonance energy transfer (FRET) was conducted to determine whether heterogeneous micelles were formed when products were mixed with Pam_2_CSK_4_. Solutions of 36 μM M2_2–16_ peptide (containing 0.1 μM FAM-M2_2–16_) or 36 μM PA (containing 5 μM Palm_2_K-K(FAM)-M2_2–16_-(KE)_4_) with or without 4 μM TAMRA-Pam_2_CSK_4_ were prepared. The ratios of FAM-labeled peptide (or PA) to unlabeled peptide (or PA) were chosen in order to produce similar intensities between peptide and PA groups. Samples were excited at 450 nm and fluorescent emissions were measured from 475 to 700 nm using a Biotek Cytation 5 Spectrophotometer.

### 2.6. Murine Immunization

In vivo studies were performed in accordance with protocol 32,204 approved by the University of Missouri Animal Care and Use Committee. Each vaccination group consisted of 4–5 female and 3–4 male BALB/c mice at 10–11 weeks of age. Vaccines were prepared by diluting the specified amount of peptide or PA (with adjuvant as applicable) in 100 μL PBS and incubated at 4 °C overnight to allow for equilibration. Vaccines consisted of 20 nmol of peptide or PA, with or without 2.22 nmol Pam_2_CSK_4_ as outlined in Table 1. On day 0, mice were immunized with a subcutaneous injection in the nape of the neck. On day 14, whole blood was collected from the saphenous veins of each mouse. An equivalent second dose of vaccine was administered to the mice on day 21. On day 35, 14 days after the second vaccination, mice were sacrificed via carbon dioxide asphyxiation, following ACUC protocol guidelines. Cardiac puncture was used as a secondary means of euthanasia and for terminal blood collection.

### 2.7. Serum Enzyme-Linked Immunosorbent Assay

Serum samples were generated by centrifuging whole blood at 9400× *g* for 10 min and collecting the supernatant, after which they were frozen at −80 °C until further use. To quantify serum antibody content, enzyme-linked immunosorbent assay (ELISA) plates were coated at 4 °C overnight with a solution of 1.69 μM M2e peptide in carbonate buffer. The following day, plates were washed with 0.05% Tween-20 in PBS (all subsequent washes used the same wash buffer), then blocked with assay diluent (10% fetal bovine serum (FBS) in PBS) for 1 h. Serum samples were initially diluted 100-fold in assay diluent on the plate and serially diluted 2-fold down twenty-one times to 209,715,200. Plates were sealed and incubated at 4 °C overnight. The next day, plates were washed, then incubated with a 3000-fold dilution of secondary antibody (horseradish peroxidase (HRP)-conjugated goat anti-mouse IgM, IgG(H&L), IgG1, IgG2a, and IgG3, Invitrogen (Waltham, MA, USA)) in assay diluent for 1 h. After washing, TMB (3,3′,5,5′ tetramethylbenzidine, BioLegend (San Diego, CA, USA)) was added to each well and allowed to incubate in the dark for 30 min before absorbance was measured at 650 nm using a Biotek Cytation 5 Spectrophotometer to visualize HRP content. Samples were normalized across plates by subtracting the absorbance of assay-diluent only wells from the absorbance of each serum-containing well. Antibody titers were calculated as the lowest serum dilution with an absorbance at least twice that of the average absorbance of the serum samples from PBS-vaccinated mice at the same serum dilution.

### 2.8. Bone Marrow-Derived Dendritic Cell Generation and Assessment

Bone marrow was harvested from the femurs and tibias of 4-month-old BALB/c mice and red blood cells were lysed using ammonium-chloride-potassium (ACK) lysing buffer. Bone marrow cells were cultured in complete RPMI media (i.e., 10% FBS, 100 U/mL penicillin, 100 μg/mL streptomycin, and 50 μM β-mercaptoethanol) with 20 ng/mL granulocyte-macrophage colony-stimulating factor (GM-CSF) to promote differentiation into bone marrow-derived dendritic cells (BMDCs). After 10 days, BMDCs were plated in untreated 24-well plates and cultured with peptide or PA for 24 h. Supernatants were collected and stored at −20 °C for later use. BMDCs were harvested and processed for flow cytometry analysis. More specifically, cells were removed from the plate, blocked with Trustain FcX anti-mouse CD16/32 antibody (BioLegend), then stained with phycoerythrin-Cyaninine7 (PE-Cy7) anti-mouse CD11c (BioLegend), fluorescein isothiocyanate (FITC) anti-mouse CD40 (BioLegend), and allophycocyanin (APC) anti-mouse MHC II (BioLegend) before being fixed with 4% para-formaldehyde. Cells were analyzed on a BD LSR Fortessa Flow Cytometer (Franklin Lakes, NJ, USA) within 48 h after fixation. Events were gated according to the strategy shown in Figure S2, with viable samples having a “valid cells” gate of at least 45% of the total events. To evaluate cytokine secretion content (i.e., TNF-α and IL-12/IL-23) in the media supernatant, ELISAs were completed according to kit instructions (BioLegend).

### 2.9. Statistics

One-way analysis of variance (ANOVA) and Tukey’s Honestly Significant Difference (HSD) tests were performed using GraphPad Prism software (Version 7.02). Within a graph, groups that possess different letters have statistically significant difference in mean (*p* ≤ 0.05) whereas those that possess the same letter have similar means (*p* > 0.05).

## 3. Results

### 3.1. M2_2–16_ and Palm_2_K-M2_2–16_-(KE)_4_ Form Small Micelles at Low Concentrations Making Them Ideal for Vaccine Applications

**Figure 1:**
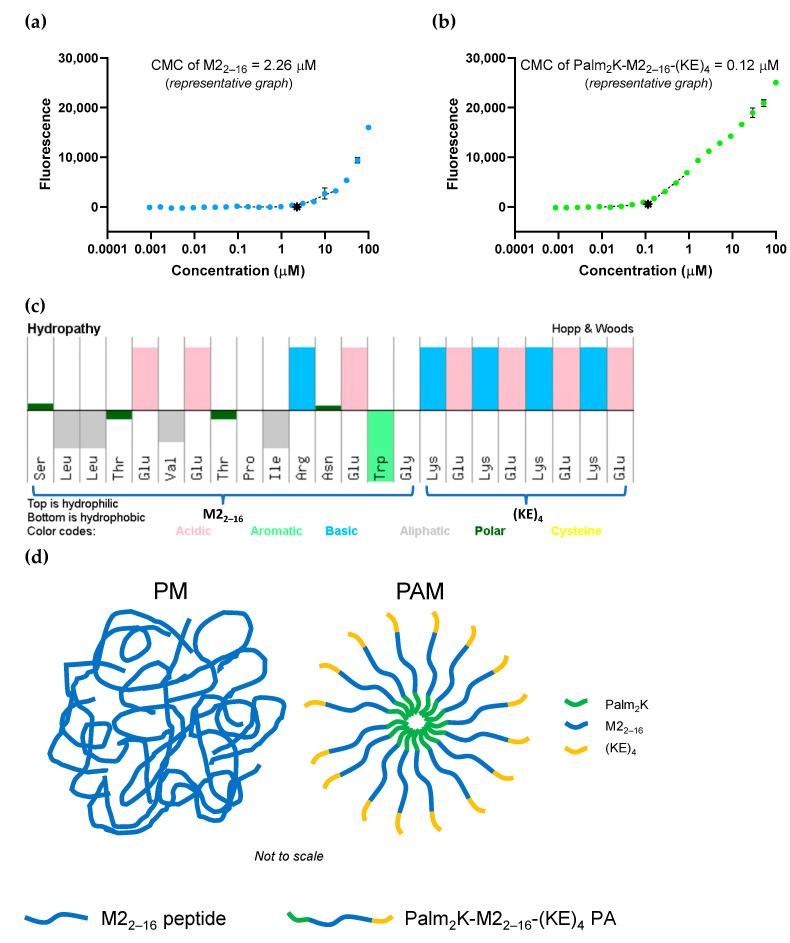
Critical micelle concentration assessment showed both M2_2–16_ peptide and Palm_2_K-M2_2–16_-(KE)_4_ peptide amphiphile readily self-assemble. (**a**) M2_2–16_ peptide unexpectedly formed micelles at 2.26 μM (∗) as shown in the representative CMC graph. (**b**) A representative CMC graph of Palm_2_K-M2_2–16_-(KE)_4_ PA showed micellization occurs as low as 0.12 μM (∗). (**c**) A hydropathy chart (reprinted from Pepcalc.com) of M2_2–16_-(KE)_4_ shows that the M2_2–16_ peptide has regions of moderate hydrophobicity and hydrophilicity, whereas the (KE)_4_ region on the C-terminus contributes the most hydrophilicity to the Palm_2_K-M2_2–16_-(KE)_4_ PA [35]. (**d**) With this in mind, weaker hydrophobic forces in the peptidyl micelles (**left**) could create more disordered micelles as compared to the distinct regions of PAs yielding more structured micelles (**right**).

Purified M2_2–16_ peptide and Palm_2_K-M2_2–16_-(KE)_4_ PA were characterized for their capacity to self-assemble using a CMC assay. Surprisingly, M2_2–16_ peptide appeared to self-assemble with a CMC of 2.70 ± 1.60 μM (Figure 1a). These peptidyl micelles (PMs) were unexpected because previous unmodified peptide controls used in various PAM-related research (e.g., OVA_BT_ and vasoactive intestinal peptide) had not been shown to possess this behavior [28,31]. This result was quite exciting as it uniquely allowed for the decoupling of the effects that lipid presence and micellization have on peptide physical properties and immunogenicity. As expected, the Palm_2_K-M2_2–16_-(KE)_4_ PA also self-assembled, having a CMC of 0.15 ± 0.06 μM (Figure 1b), uncovering that PAMs possess an order of magnitude lower CMC compared to analogous PMs. To probe the driving force behind PM micellization, CMCs of M2_2–16_ peptide were conducted at various salt concentrations (Milli-Q deionized, distilled water (ddH_2_O) and 10x PBS) and pHs (ddH_2_O at pH ~ 3 and pH ~ 13). Interestingly, altering salt concentration or pH did not appreciably affect the self-assembly of M2_2–16_ peptide with CMCs ranging from 0.56–5.29 μM determined under these conditions (Figure S3). As ion content and amino acid side chain charge greatly influences electrostatic complexation [32,33], these results indicate that M2_2–16_ micellization was likely hydrophobically driven. When considering the hydropathy of the M2_2–16_ peptide and the Palm_2_K-M2_2–16_-(KE)_4_ PA (Figure 1c), the difference in CMCs between the PMs and PAMs becomes clearer. The hydrophobic forces involved in the self-assembly of PMs were most likely weaker than those in PAMs because the hydrophobic and hydrophilic domains were more interspersed in the peptide than the PA leading to a somewhat disordered aggregate structure in PMs (Figure 1d). In contrast, the PAs were strongly amphipathic with distinct regions of a very hydrophobic dipalmitoyllysine on the N-terminus and a very hydrophilic charge block on the C-terminus triggering micellization at a lower concentration in solution [34].

**Figure 2:**
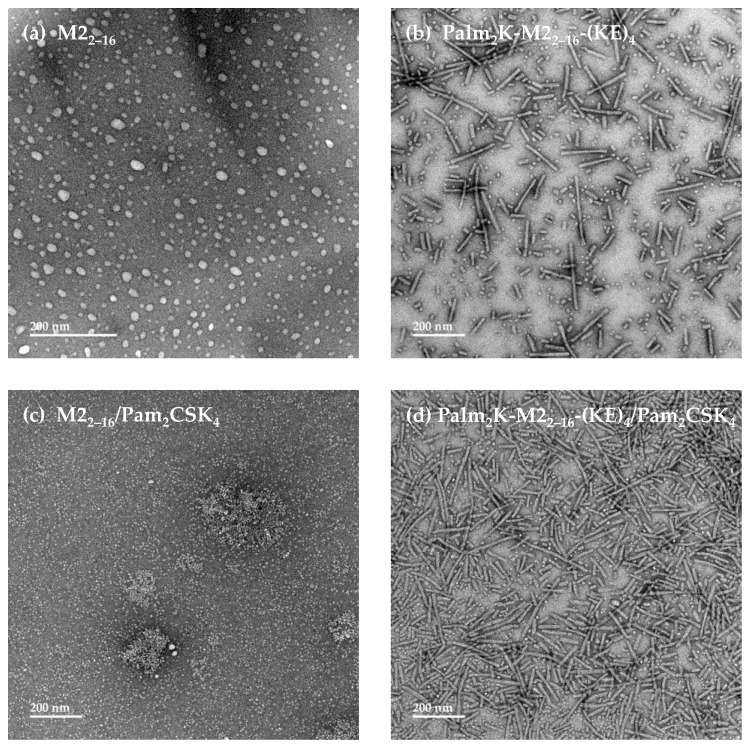
Transmission electron microscopy showed that M2_2–16_ peptide and Palm_2_K-M2_2–16_-(KE)_4_ peptide amphiphile formed small spherical and cylindrical micelles, respectively, for which the addition of Pam_2_CSK_4_ had differential effects on their morphology. (**a**) TEM confirmed M2_2–16_ peptide self-assembled into small spherical micelles (~10–25 nm in diameter). (**b**) Palm_2_K-M2_2–16_-(KE)_4_ PA formed moderately short cylindrical micelles (~10 nm in diameter by 100–200 nm in length). (**c**) The addition of 10% Pam_2_CSK_4_ to 90% M2_2–16_ peptide changed micelle morphology to smaller spheres and very short cylinders. (**d**) The combination of 10% Pam_2_CSK_4_ and 90% Palm_2_K-M2_2–16_-(KE)_4_ PA produced a mix of spherical and short cylindrical micelles, hardly impacting micelle size and shape at all.

To assess micelle morphology, both M2_2–16_ peptide and Palm_2_K-M2_2–16_-(KE)_4_ PA were visualized using TEM at a concentration above the CMC (i.e., 20 μM). Micrographs of M2_2–16_ distinctly show the formation of spherical micelles approximately 10–25 nm in diameter (Figure 2a). Interestingly, Palm_2_K-M2_2–16_-(KE)_4_ was found to produce mostly moderately short cylindrical micelles approximately 10 nm in diameter and 100–200 nm in length (Figure 2b). The modest shape difference observed between M2_2–16_ PMs and Palm_2_K-M2_2–16_-(KE)_4_ PAMs is likely a byproduct of the difference in the amphiphilicity of their constituent unimers. The more distinct hydrophobic and hydrophilic regions of PAs allow for them to pack in a more ordered, and, therefore, tighter confirmation, leading to a narrower unimer cone shape better favoring the formation of cylindrical structures over spherical ones. In contrast, the weaker definition around the hydrophobic and hydrophilic regions of the M2_2–16_ peptide likely leads to wider cones and the tendency to form spherical micelles (Figure 1d).

As we were interested in studying the immunological impact of micellar adjuvant incorporation, the known TLR2 agonist and lipopeptide Pam_2_CSK_4_ was explored for its influence on PM and PAM formation. Pam_2_CSK_4_ alone was found to self-assemble into mostly small spherical micelles less than 10 nm in diameter with some occasional cylindrical micelles formed (Figure S4), which aligned with what has been seen in the literature [36]. M2_2–16_ peptide or Palm_2_K-M2_2–16_-(KE)_4_ PA were then mixed with Pam_2_CSK_4_ at a 90/10 ratio and observed using TEM to assess the influence of adjuvant incorporation on micelle shape. For M2_2–16_ PMs, the presence of Pam_2_CSK_4_ eliminated the formation of micelles larger than 10 nm in diameter while preserving their spherical morphology (Figure 2c). In contrast, heterogeneous Palm_2_K-M2_2–16_-(KE)_4_/Pam_2_CSK_4_ PAMs appeared to be mostly cylindrical in shape (Figure 2d), quite similar to Palm_2_K-M2_2–16_-(KE)_4_ PAMs alone. Even though micellization of PMs and PAMs both seemed to be hydrophobically driven, it is not entirely unsurprising that there were different responses when Pam_2_CSK_4_ was added, given the differences in amphipathicity between M2_2–16_ peptide and Palm_2_K-M2_2–16_-(KE)_4_ PA. The M2_2–16_ peptide alone does not have as strong of a hydrophobic element as the N-terminal dipalimtoyllysine of Palm_2_K-M2_2–16_-(KE)_4_, so despite the fact that M2_2–16_ PM micellization was driven by hydrophobic forces, it is possible that the morphology of the M2_2–16_/Pam_2_CSK_4_ PMs was at least partially dictated by electrostatic forces. Specifically, the change in micelle size from M2_2–16_ to M2_2–16_/Pam_2_CSK_4_ could be explained by the repulsion of negatively charged residues in M2_2–16_ peptide being disrupted and replaced by tight complexation with the positively charged lysines in Pam_2_CSK_4_, thus resulting in the formation of smaller micelles [37]. In the case of the PAMs, however, Palm_2_K-M2_2–16_-(KE)_4_ and Pam_2_CSK_4_ are structurally similar as both are lipopeptides with two 16-carbon tails and charged residues on their C-terminus. Therefore, it is unsurprising that there were no substantial morphological changes to Palm_2_K-M2_2–16_-(KE)_4_ PAMs with the addition of Pam_2_CSK_4_.

**Figure 3:**
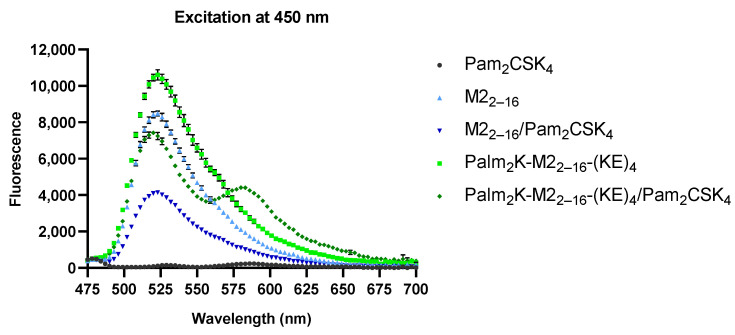
Changes to the fluorescence spectra at 525 nm of the peptides and peptide amphiphiles after adding Pam_2_CSK_4_ confirmed that Pam_2_CSK_4_ was integrated into both peptidyl micelles and peptide amphiphile micelles.

To confirm that Pam_2_CSK_4_ integrated into the PMs and PAMs, FAM-labeled peptide or PA was mixed with TAMRA-labeled Pam_2_CSK_4_. The fluorophores FAM and TAMRA are a FRET pair, where the energy emitted by FAM can be absorbed by TAMRA, resulting in TAMRA emission to be observed at 580 nm when in close physical proximity to FAM. FRET was clearly observed in the PAMs, as a decrease in fluorescence intensity at 525 nm (i.e., the FAM emission wavelength) corresponded to the appearance of a local fluorescence maximum at 580 nm (i.e., the TAMRA emission wavelength) when TAMRA-Pam_2_CSK_4_ was present, confirming the presence of heterogeneous micelles (Figure 3). While the M2_2–16_ PMs did not exhibit a local fluorescence maximum at 580 nm, there was a substantial decrease in fluorescence intensity at 525 nm when in the presence of TAMRA-Pam_2_CSK_4_, which suggested that there was still FRET and, consequently, that M2_2–16_ and Pam_2_CSK_4_ formed heterogenous micelles. To provide further evidence of FRET in the M2_2–16_ PMs, the fluorescence of increasing ratios of TAMRA-Pam_2_CSK_4_ to M2_2–16_ peptide was measured. With increasing concentrations of TAMRA-Pam_2_CSK_4_ a local maximum at 580 nm appeared, validating the existence of FRET in the PMs (Figure S5).

**Figure 4:**
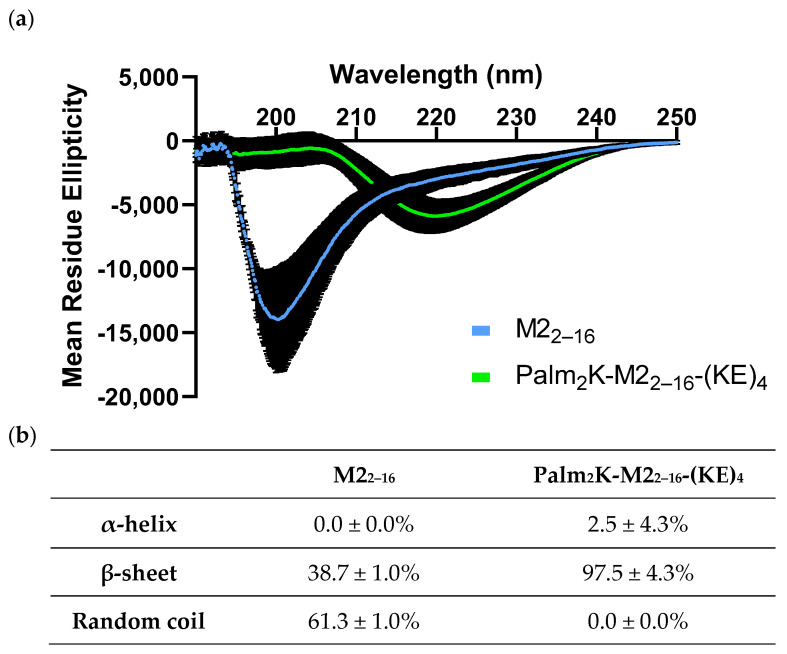
Peptidyl micelles and peptide amphiphile micelles had distinctly different secondary structures. (**a**) Circular dichroism (CD) spectra of M2_2–16_ PMs and Palm_2_K-M2_2–16_-(KE)_4_ PAMs showed minima at 200 nm and 219 nm, respectively, and Palm_2_K-M2_2–16_-(KE)_4_ PAMs had a maximum at 204 nm. (**b**) Based on reference CD data for poly(lysine) and poly(glutamine), the estimated secondary structure of M2_2–16_ PMs was mostly random coil whereas Palm_2_K-M2_2–16_-(KE)_4_ PAMs were almost entirely β-sheet.

Given the morphological differences between M2_2–16_ PMs and Palm_2_K-M2_2–16_-(KE)_4_ PAMs, circular dichroism (CD) analysis was carried out to evaluate whether those dissimilarities influenced peptide secondary structure (Figure 4). When fit to known spectra, PMs were found to be mostly random coil (61.3%) in nature, as shown by a minimum at 198 nm, but they also had considerable β-sheet character (38.7%). PAMs, on the other hand, were determined to be almost entirely β-sheet (i.e., 97.5%), as shown by a minimum at 219 nm and a maximum at 205 nm. The structure of the M2 protein ectodomain in the context of the influenza virus has not been well characterized, except when untethered from the rest of the protein and bound to antibodies or other entities. Published experimental results and computational modeling using predictive software (PEP-FOLD 3 and I-TASSER (Version 5.2)) have been inconsistent, showing the presence of some α-helical or β-sheet character or random coil alone [38,39,40,41]. Although there was not a clear expectation of what the desired secondary structure composition should be, Qiao and colleagues found that polar regions with more constrained secondary structure (e.g., helices or sheets) on the surface of a protein were more favorable for antibody recognition when a library of 350 antigen-antibody complexes were analyzed [42].

### 3.2. Peptide Amphiphile Micelles Elicited Strong Antibody Titers After the Primary Immunization, but Both Micelle Types Elicited Strong IgG Titers After the Booster

**Figure 5:**
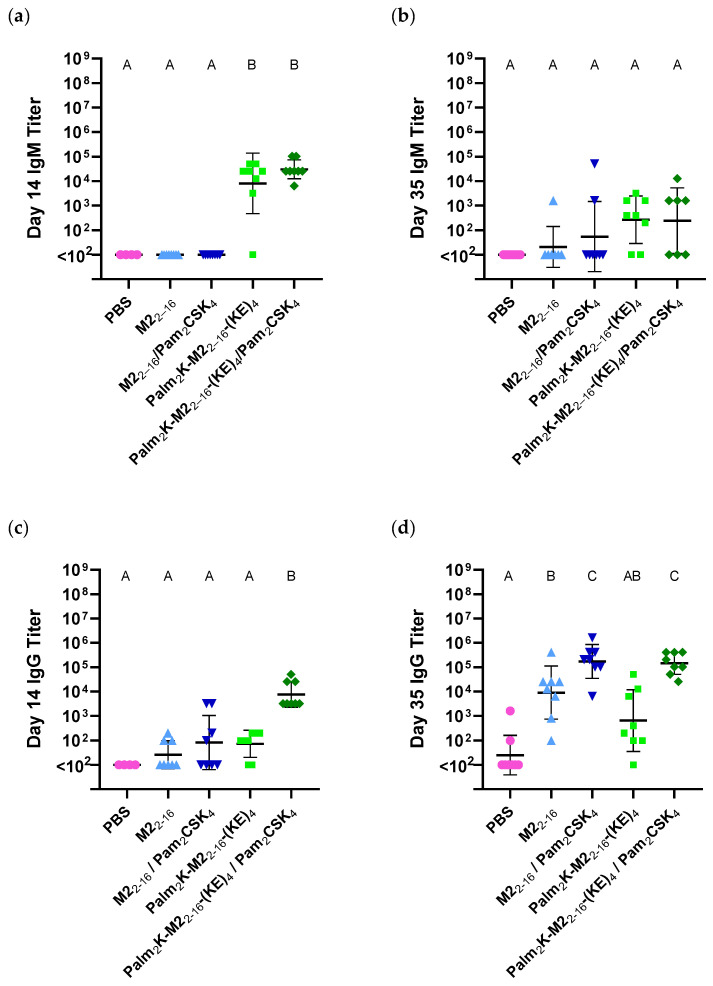
Serum antibody titers were higher from mice given a single dose of peptide amphiphile micelles than those given peptidyl micelles, while prime-boost responses were primarily driven by co-delivery with Pam_2_CSK_4_. (**a**) PAMs induced a strong IgM response at day 14, regardless of adjuvant incorporation whereas PMs did not induce above background IgM production. (**b**) No significant differences were found in IgM titers between any vaccine groups at day 35, although those induced by PAMs were slightly higher than background, but less than what were observed at day 14. (**c**) Only Palm_2_K-M2_2–16_-(KE)_4_/Pam_2_CSK_4_ induced IgG titers at day 14 above baseline. (**d**) All micelle groups elicited IgG titers above baseline in day 35 serum, though Palm_2_K-M2_2–16_-(KE)_4_ was unable to do so with statistical significance. Pam_2_CSK_4_-containing groups elicited statistically significantly elevated titers over groups without adjuvant. Within a graph, groups that possess different letters have statistically significant difference in mean (*p* ≤ 0.05) whereas those that possess the same letter have similar means (*p* > 0.05).

To evaluate the immune response against both micelle types, mice were subcutaneously vaccinated with either formulation (with or without the adjuvant Pam_2_CSK_4_). Blood was collected at days 14 and 35 (i.e., 14 days after the first and second vaccinations, respectively). ELISAs were conducted with the serum from collected blood to evaluate antibody production against the vaccines. Interestingly, at day 14, there were no IgM responders in either PM group (with or without adjuvant), but both PAM groups had similar statistically significantly higher titers over the PBS vaccine baseline, independent of the presence of Pam_2_CSK_4_ (Figure 5a). By day 35, although there were no longer statistically significant differences between any groups, there were still several more responders in the PAM vaccine groups (10 total) compared to the PM vaccine groups (3 total), though PAM-induced IgM titers did seem to have slightly decreased from those at day 14 (Figure 5b). Strong IgM responses (relative to soluble peptide antigens) have been documented in PAMs [30,43], liposomes [44,45], and even PMs [46]. The significant IgM titers suggested a T cell-independent immune response mechanism, which, although typically associated with polysaccharide antigens, can be utilized by multivalent peptide antigens to directly activate B cells without requiring a signal from helper T cells [47,48]. Although no significant IgM responses were observed in PMs in this work, this disagreement with literature could potentially be explained by structural differences between the M2_2–16_ PMs and the O-Q11 PMs studied in Rudra et al. [46] In the case of O-Q11, the OVA antigen was covalently attached to the Q11 self-assembling peptide, which constituted the core of the cylindrical micelle, allowing OVA to be more uniformly displayed on the outside of the micelle more closely resembling the morphology of the Palm_2_K-M2_2–16_-(KE)_4_ PAMs used in this work. M2_2–16_ PMs, on the other hand, formed spherical micelles of varying diameters with much lower β-sheet conformation, suggesting a less ordered display of the M2_2–16_ antigen, as depicted in the cartoon in Figure 1d. Antigen display has been theorized to directly affect the antibody isotype response, which could explain the difference in IgM response between PAMs and PMs in this work [44,49,50]. Specifically, Therien et al. showed that an antigen covalently linked to the surface of liposomes elicited a response with a lower IgG/IgM ratio (i.e., a response more skewed towards IgM) compared to antigens encapsulated within liposomes [44].

Day 14 IgG titers showed a few responders in the mice vaccinated with M2_2–16_, M2_2–16_/Pam_2_CSK_4_, and Palm_2_K-M2_2–16_-(KE)_4_, but when combined with non-responders, these results were found to be statistically insignificantly above background (Figure 5c). Excitingly, however, the adjuvant-loaded PAM formulation (i.e., Palm_2_K-M2_2–16_-(KE)_4_/Pam_2_CSK_4_) induced statistically appreciable IgG content at day 14, suggesting its potential utility as a single-dose vaccine. This is especially relevant for vaccines against rapidly emerging or seasonal pathogens like COVID-19 or influenza, respectively, in which a single-dose regimen could improve herd immunity in cases of limited vaccine supply (such as during pandemics), as well as decrease costs and increase vaccine accessibility for people with intermittent access to healthcare [51,52]. The day 35 IgG data showed that both adjuvant-free micelle formulations (i.e., M2_2–16_ and Palm_2_K-M2_2–16_-(KE)_4_) induced an antibody response though interestingly PMs induced slightly higher, though statistically insignificant, titers above PAMs (Figure 5d). The incorporation of adjuvant in either PMs or PAMs yielded vaccines that induced substantially elevated IgG titers in which differences between the micelle-type were minimal, suggesting that stronger immunity can be achieved for any formulation with a multi-dose regimen.

**Figure 6:**
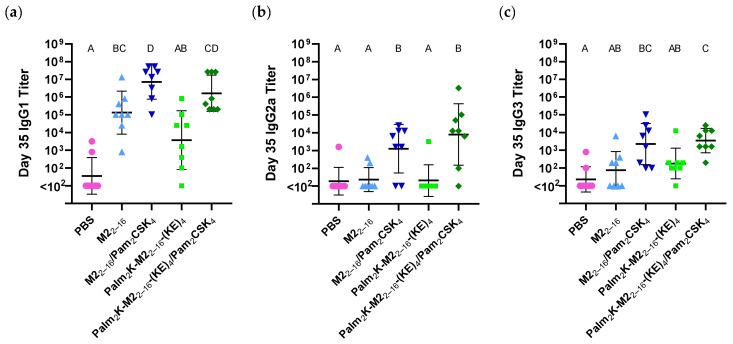
Immunoglobulin subtype assessment showed an IgG1-skewed antibody response for peptidyl and peptide amphiphile micelles. (**a**) IgG1 titers were highest in Pam_2_CSK_4_-containing vaccine groups, with PMs having slightly (but not significantly) higher titers than the PAMs. (**b**) IgG2a titers were lower than IgG1 titers, with only adjuvant-containing groups above baseline. Although not statistically significant, Palm_2_K-M2_2–16_-(KE)_4_/Pam_2_CSK_4_ had a slightly higher titer than M2_2–16_/Pam_2_CSK_4_. (**c**) IgG3 titers matched the IgG1 titers trend though much lower in magnitude. Micelles without adjuvant were above baseline, but not with statistical significance. Both PAMs and PMs with adjuvant elicited titers above baseline, but only the Palm_2_K-M2_2–16_-(KE)_4_/Pam_2_CSK_4_ titer was statistically significantly higher than titers generated by micelle formulations without adjuvant. Within a graph, groups that possess different letters have statistically significant difference in mean (*p* ≤ 0.05) whereas those that possess the same letter have similar means (*p* > 0.05).

To further investigate the antibody response, ELISAs were conducted on the serum collected from day 35 to measure the relative amounts present of various IgG subclasses (i.e., IgG1, IgG2a, and IgG3). IgG1 titers (Figure 6a) largely aligned with total IgG titers (Figure 5d), in that titers were highest in Pam_2_CSK_4_-containing vaccine groups and that the non-adjuvanted PMs had a slightly higher (although statistically insignificant) titer than the non-adjuvanted PAMs. The PAMs without any adjuvant also did not produce a titer statistically above baseline. For IgG2a titers, only the PMs and PAMs with adjuvant elicited titers statistically above baseline (and the majority of serum samples in the groups without adjuvant were non-responders) (Figure 6b). IgG3 titers largely aligned with IgG2a titers, except that there were more positive responders in all groups (Figure 6c).

IgG1, especially in the formulations without adjuvant, was the primary IgG subclass generated in response to all vaccine formulations, complemented with lower IgG2a and IgG3 responses. This aligned with several previous studies that have used PAMs containing different antigens [29,30,43]. Ideally, the influenza vaccine response would mimic the natural host response to better prepare the immune system for later exposure to the virus. In mice, IgG2a is the predominant antibody generated in the immune response against influenza infection, followed by IgG1, then IgG3 [53]. While the IgG subtype ratio was not IgG2a-dominant in this study, the administration route of the vaccine could have played a prominent role in the observed outcome. Specifically, Wareing and colleagues compared the generation of IgA and IgG2a antibodies for an influenza vaccine delivered via different routes of administration (i.e., subcutaneous, intramuscular, and intranasal) [54]. They found that intranasal delivery produced the greatest amount of IgA antibodies in the lungs and the highest serum IgG2a titers. To our knowledge, PAM vaccines have not been administered intranasally to date, but this could be a worthwhile approach to investigate to try to produce a serum IgG2a-focused antibody profile in the future, specifically for application as an influenza vaccine.

### 3.3. Bone Marrow-Derived Dendritic Cells Were Activated by Adjuvant-Supplemented Peptidyl Micelles and Peptide Amphiphile Micelles

**Figure 7:**
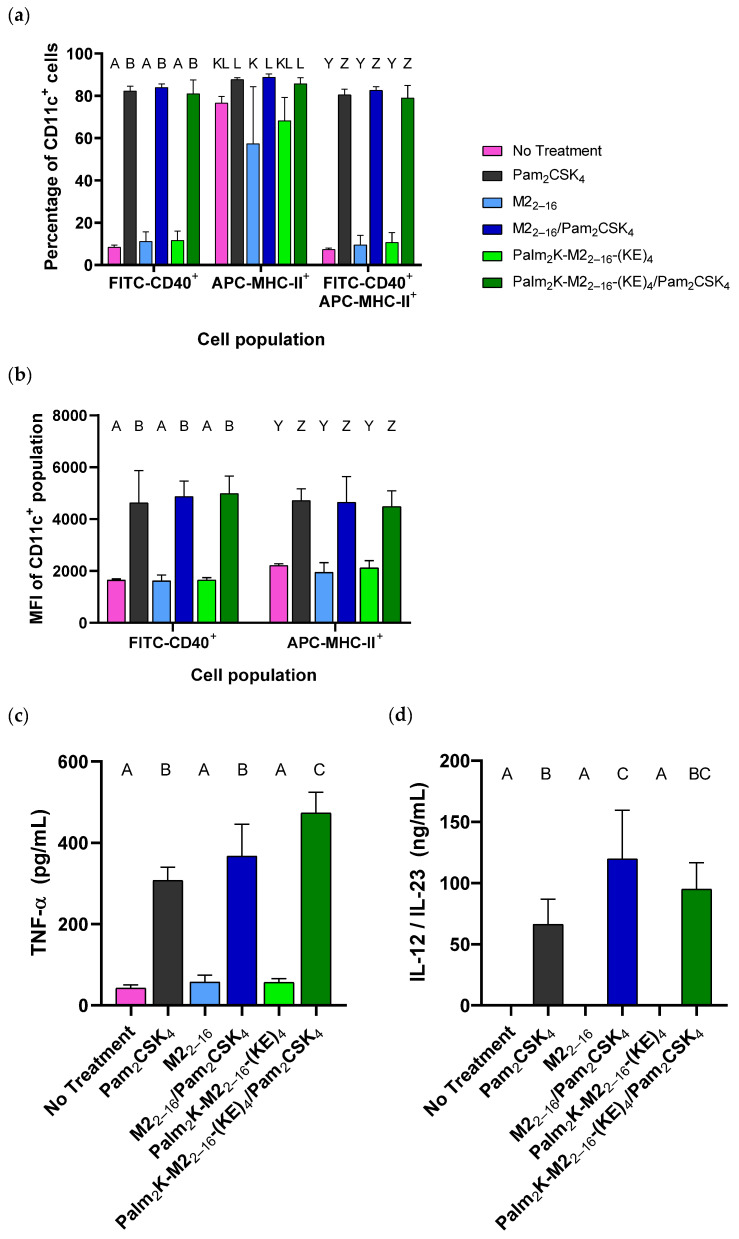
Bone marrow-derived dendritic cell activation is primarily affected by the presence of Pam_2_CSK_4._ (**a**) The percentage of CD11c^+^ cells expressing CD40 (but not MHC-II) increased significantly when treated with Pam_2_CSK_4_-containing formulations. (**b**) Treatments containing Pam_2_CSK_4_ strengthened the MFIs of the FITC and APC channels for CD11c^+^CD40^+^ and CD11c^+^MHC-II^+^ cell populations, respectively. Pam_2_CSK_4_ elicited higher production of proinflammatory cytokines (**c**) TNF-α and (**d**) IL-12/IL-23, as determined by ELISAs. Groups that possess different letters have statistically significant differences in mean (*p* ≤ 0.05), whereas those that possess the same letter have similar means (*p* > 0.05). Statistical comparisons were only made between different peptide treatments within the same channel or gating scheme.

To test the innate immunogenicity of the micelle formulations, bone marrow-derived dendritic cells (BMDCs) were cultured and treated with 1.8 μM antigen-containing micelles (PMs or PAMs) with or without 0.2 μM Pam_2_CSK_4_ for 24 h. BMDCs were evaluated using flow cytometry and identified by CD11c expression with the gating strategy illustrated in Figure S2. BMDC activation was identified by elevated CD40 and MHC-II expression. The percent of CD11c^+^ cells expressing CD40 was significantly increased among cells exposed to all treatments containing adjuvant (i.e., Pam_2_CSK_4_ alone or with either micelle type) without measurable differences between those groups (Figure 7a). There was less of an effect (not statistically significant) for MHC-II expression, although the mean cell percentages for CD11c^+^MHC-II^+^ cells in the Pam_2_CSK_4_-treated groups were higher than in groups not treated with Pam_2_CSK_4_. This was likely due to the fact that MHC-II is constitutively expressed on dendritic cells and further upregulated upon activation [55]. The adjuvant effect was still present when comparing cell data between peptide treatments in the CD11c^+^ population expressing both activation markers (CD40 and MHC-II), although this was likely a carry-over effect from the significant differences between groups in the CD40^+^ population. When looking at the median fluorescence intensity (MFI) of FITC-CD40 among CD40^+^CD11c^+^ cells, there was again a distinct though similar signal enhancement with Pam_2_CSK_4_-treated groups when compared to groups treated with PMs or PAMs alone (Figure 7b). This enhancement was also seen in the MFI of APC-MHC-II among MHC-II^+^CD11c^+^ cells despite there not being any significant effect on the percentage of MHC-II^+^ cells. This suggests that though the percentage of cells expressing MHC-II is largely unaffected, the overall expression of MHC-II was upregulated among MHC-II^+^ cells in response to adjuvant exposure. Altogether, these data support that BMDC activation was primarily driven by the presence of Pam_2_CSK_4_.

Sandwich ELISAs were conducted on supernatant media collected from treated BMDCs to evaluate their secretion of the pro-inflammatory cytokines TNF-α and IL-12/IL-23 (by quantifying the subunit p40). TNF-α and IL-12/IL-23 secretion from BMDCs (Figure 7c,d) were most dramatically impacted by the presence of Pam_2_CSK_4_, similar to the flow cytometry data. These data align with previously published work that showed that Pam_2_CSK_4_ triggers TNF-α production via the TLR-2-MyD88-NF-κB pathway [56,57] and upregulates IL-12 expression in BMDCs [58,59,60]. Given the role dendritic cells play in antigen presentation and lymphocyte activation [55], it is highly likely that there is a direct relationship between the in vitro BMDC activation data and in vivo antibody titer data.

## 4. Conclusions

Excitingly, the M2_2–16_ peptide antigen micellized on its own, an effect not previously studied, which enabled the unique opportunity to decouple the effects of micellization and antigen modification on the immune response of a micellized peptide vaccine. The addition of an N-terminal dipalmitoyllysine and a C-terminal zwitterion-like charge block to the M2_2–16_ peptide produced short cylindrical micelles, similar to prior research, with increased β-sheet character and lower CMCs when compared to the unmodified M2_2–16_ peptidyl micelles. These changes in physical characteristics corresponded with moderate changes in the immune response. Specifically, although the overall IgG response was comparable between PMs and PAMs, the PAM formulation elicited significantly higher IgM and IgG titers in the initial antibody response at day 14 and even slightly (although statistically insignificant) higher IgM titers than the PMs at day 35. While adjuvant-loaded PMs seemed to produce a slightly improved immune response in the context of the prime-booster vaccine regimen, it appears that adjuvant-loaded PAMs may be better candidates for single-dose vaccines.

## Figures and Tables

**Table 1:** Vaccine formulations used for the in vivo murine immunization study.

Vaccine Group	Vaccine Dosage
PBS	None
M2_2–16_	20 nmol M2_2–16_
M2_2–16_/Pam_2_CSK_4_	20 nmol M2_2–16_ and 2.22 nmol Pam_2_CSK_4_
Palm_2_K-M2_2–16_-(KE)_4_	20 nmol Palm_2_K-M2_2–16_-(KE)_4_
Palm_2_K-M2_2–16_-(KE)_4_/Pam_2_CSK_4_	20 nmol Palm_2_K-M2_2–16_-(KE)_4_ and 2.22 nmol Pam_2_CSK_4_

where Palm_2_K is dipalmitoyllysine and M2_2–16_ is the peptide sequence SLLTEVETPIRNEWG.

## Data Availability

All data are contained within the article and Supplementary Materials.

## Supplementary Materials

The following supporting information can be downloaded at: https://www.mdpi.com/article/10.3390/ph17111503/s1, Figure S1: Peptides were purified to greater than 90% purity using LC-MS; Figure S2: Bone marrow-derived dendritic cells were gated using the strategy illustrated above; Figure S3: Micellization of M22-16 peptide was not impacted by varying salt concentration nor pH; Figure S4: Pam2CSK4 formed mostly (a) small spherical micelles (~10 nm in diameter) and (b) occasional cylindrical micelles with variable aspect ratios; Figure S5: Fluorescence spectra of M2_2-16_ micelles with differing concentrations of TAMRA-Pam_2_CSK_4_ suggested micelle heterogeneity.
