# Supplementary material for: M2e-Derived Peptidyl and Peptide Amphiphile Micelles as Novel Influenza Vaccines
Source: Pharmaceuticals (Basel). 2024 Nov 8;17(11):1503. doi: 10.3390/ph17111503 (PMC11597048; doi:10.3390/ph17111503)
Supplement: Supplementary file 1 [file pharmaceuticals-17-01503-s001.zip › pharmaceuticals-3257325-supplementary.pdf]

pharmaceuticals

MDPI

Supplementary Materials

(a) M2$_{2-16}$

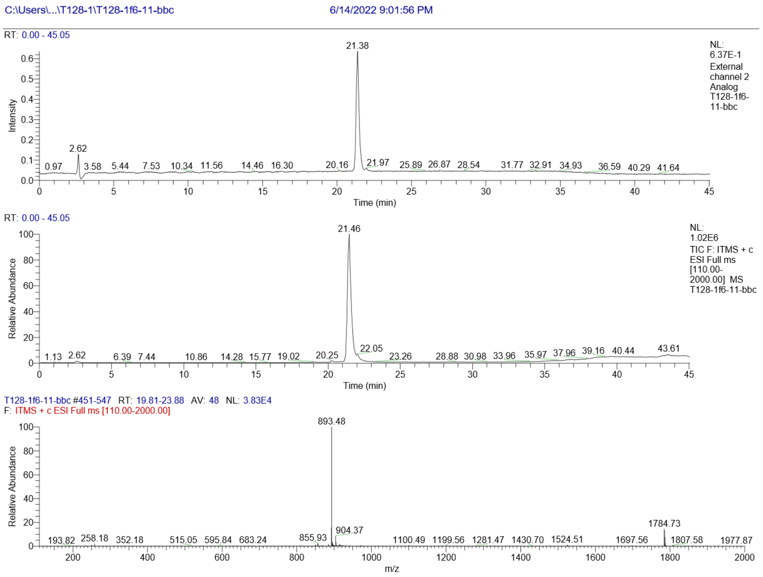

(b) Palm$_2$K-M2$_{2-16}$-(KE)$_4$

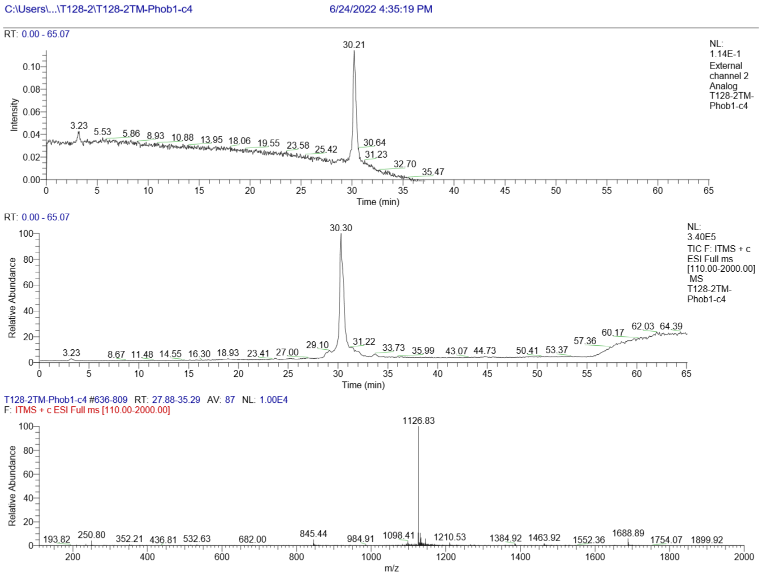

(c) M2e

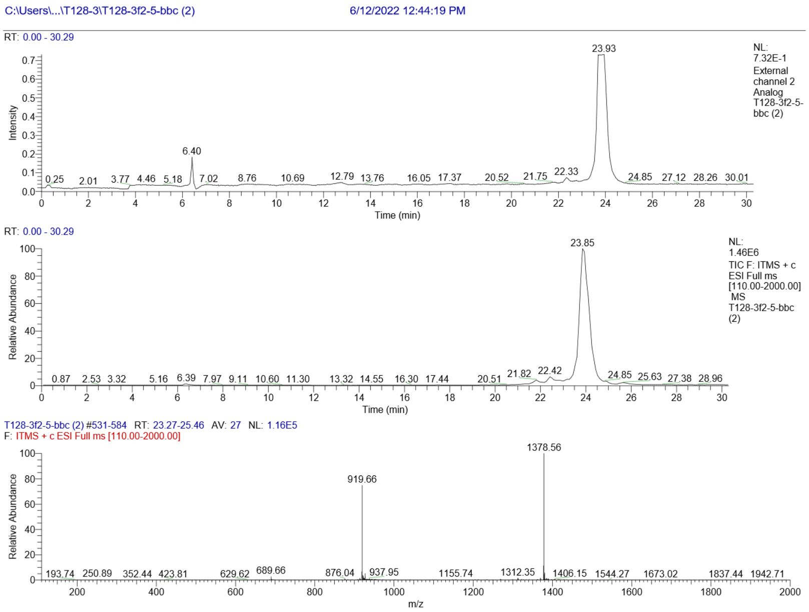

(d) FAM-M2$_{2–16}$

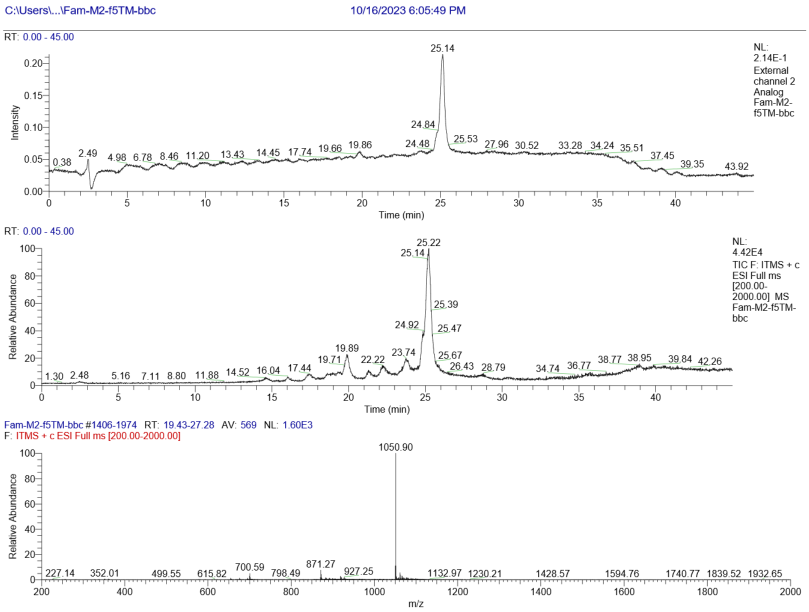

(e) Palm$_2$KK(FAM)-M2$_{2–16}$-(KE)$_4$

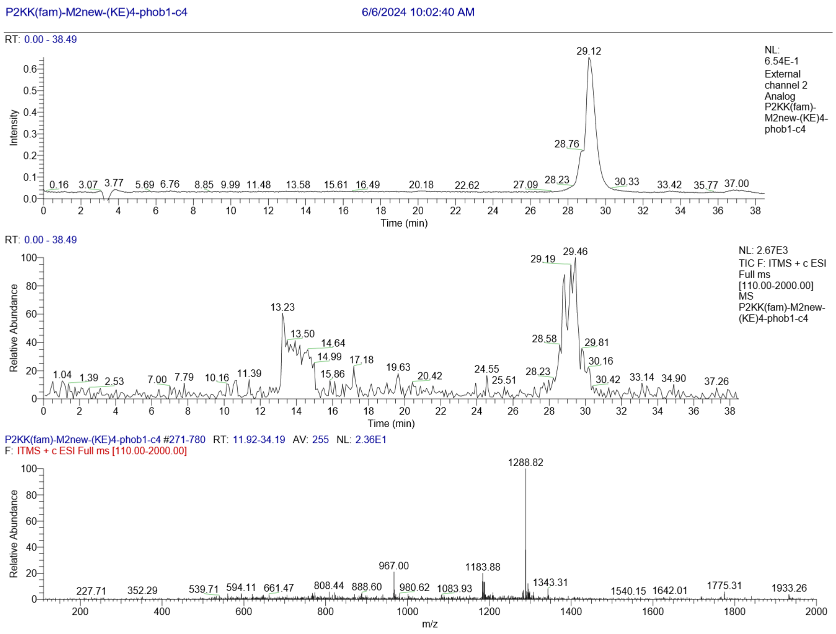

(f) TAMRA-Pam$_2$CSK$_4$

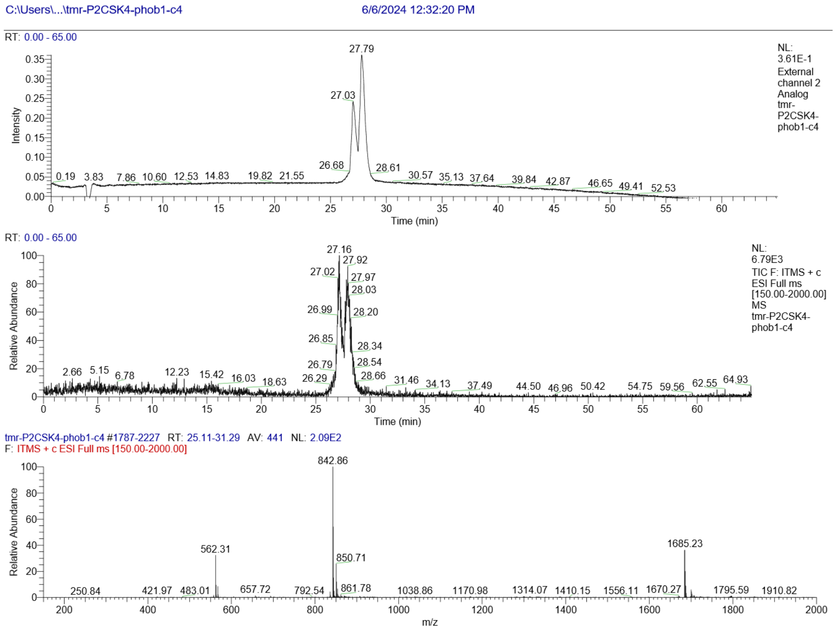

Figure S1. Peptides were purified to greater than 90% purity using LC-MS. LC-MS analyses are shown for purified (a) M2$_{2–16}$, (b) Palm$_2$K-M2$_{2–16}$-(KE)$_4$, (c) M2e, (d) FAM-M2$_{2–16}$, (e) Palm$_2$K-K(FAM)-M2$_{2–16}$-(KE)$_4$, and (f) TAMRA-Pam$_2$CSK$_4$. Top (where applicable), middle, and bottom figures of each panel consist of a UV chromatograph, a total ion count, and a mass spectrum, respectively.

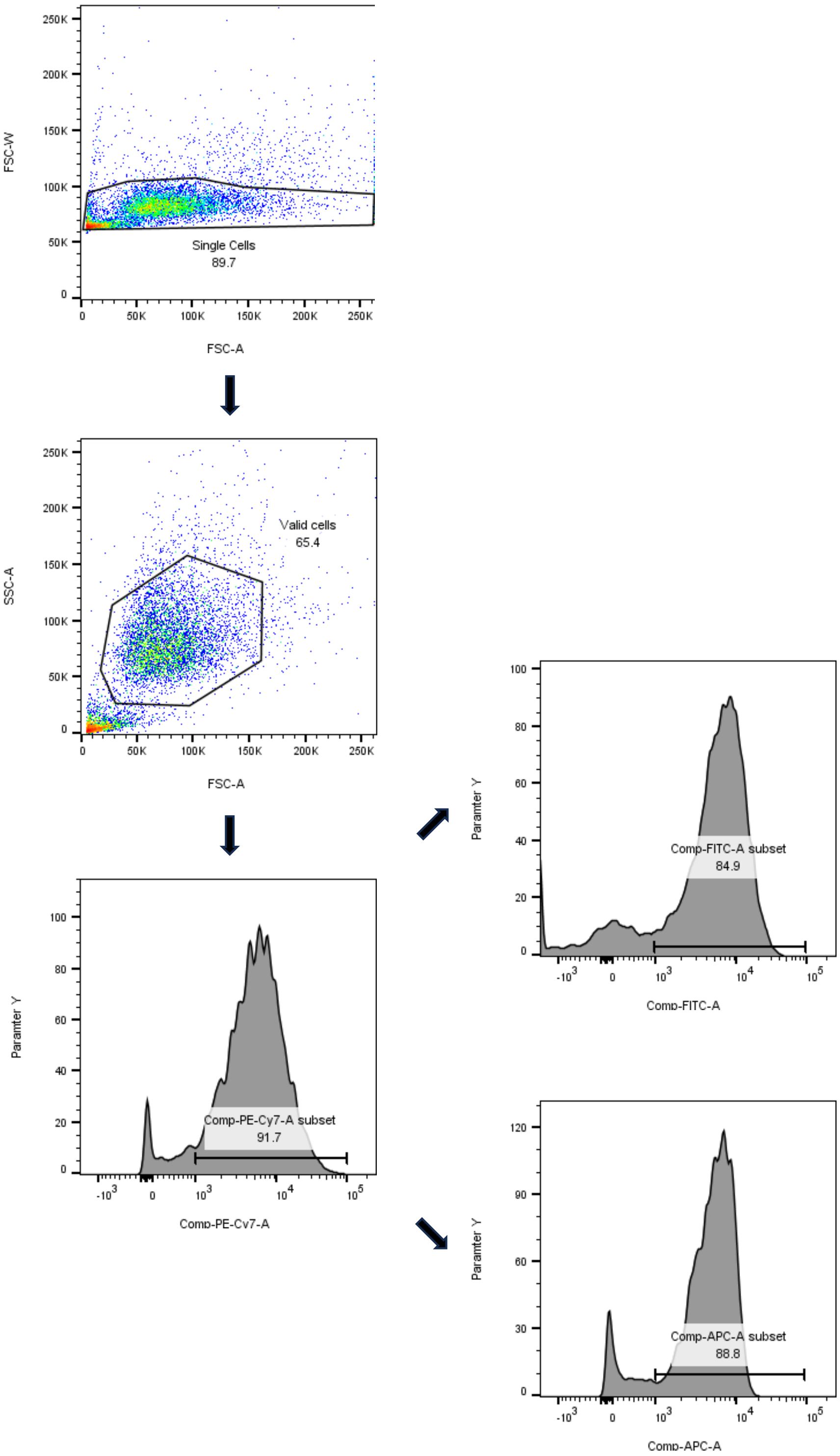

Figure S2. Bone marrow-derived dendritic cells were gated using the strategy illustrated above. Forward scatter (FSC-A vs FSC-W) was used to isolate out single cells (removing cell aggregates). Coloring indicates event density. Then cell debris was removed using FSC vs SSC (side scatter). Dendritic cells were identified by CD11c expression (PE-Cy7). Then cell activation was measured by CD40 (FITC) and MHC-II (APC) expression. Flow cytometry data was processed using FlowJo software.

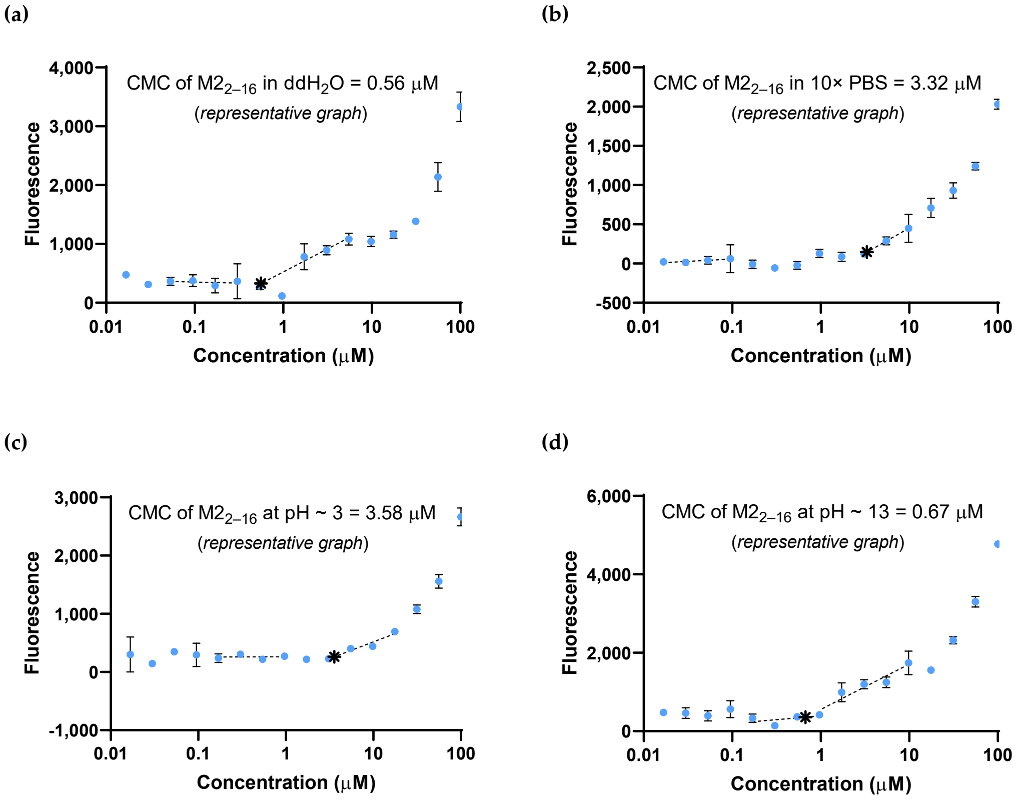

(e)

CMC Conditions	Average CMC ± Std Dev (μM)
ddH$_2$O	1.47 ± 1.10
10x PBS	2.97 ± 0.63
ddH$_2$O at pH ~ 3	3.96 ± 1.19
ddH$_2$O at pH ~ 13	2.42 ± 1.87

Figure S3. Micellization of M2$_{2-16}$ peptide was not impacted by varying salt concentration nor pH. Representative CMC graphs are shown of M2$_{2-16}$ peptide in (a) ddH$_2$O, (b) 10x PBS, (c) ddH$_2$O at pH ~ 3, and (d) ddH$_2$O at pH ~ 13. CMCs are denoted by an asterisk (*). (e) CMCs of M2$_{2-16}$ peptide in the specified solution conditions are reported as the average ± standard deviation.

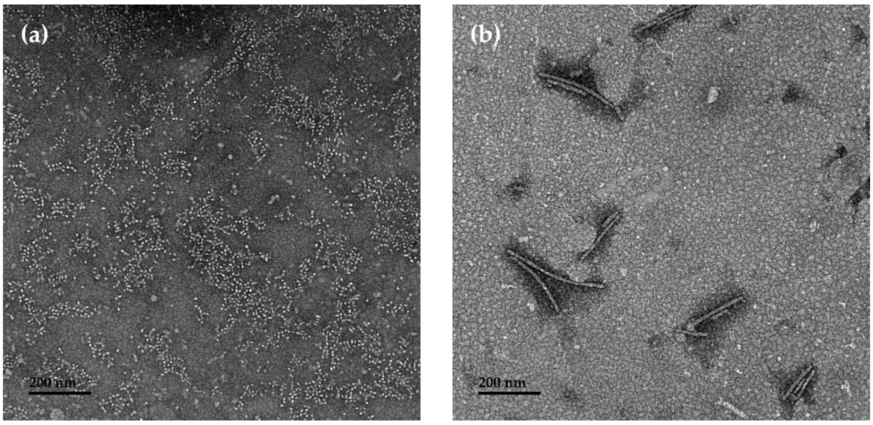

Figure S4. Pam$_2$CSK$_4$ formed mostly (a) small spherical micelles (~ 10 nm in diameter) and (b) occasional cylindrical micelles with variable aspect ratios.

(a)

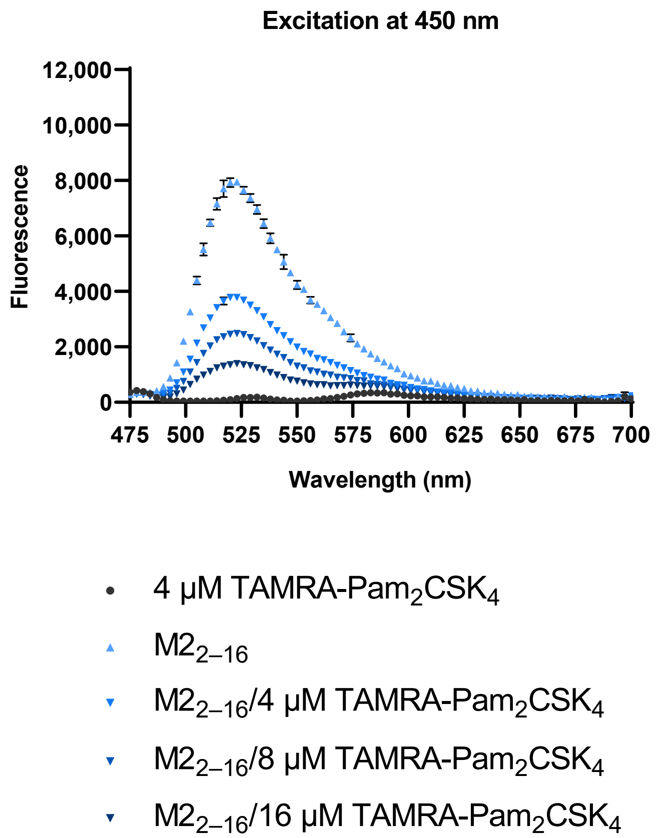

(b)

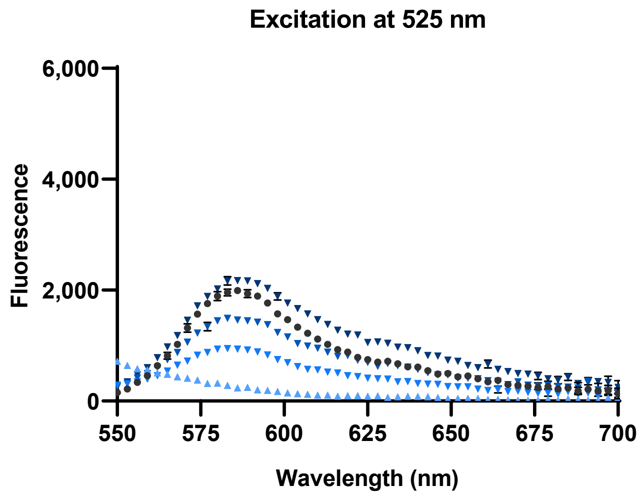

Figure S5. Fluorescence spectra of $M2_{2\text{–}16}$ micelles with differing concentrations of TAMRA-Pam_2CSK_4 suggested micelle heterogeneity. (a) Fluorescence decreased at 525 nm with increasing TAMRA-Pam_2CSK_4 concentration when excited at 450 nm (i.e., the FAM excitation wavelength). At higher concentrations of TAMRA-Pam_2CSK_4, a local maximum at 580 nm appeared (i.e., the TAMRA emission wavelength). (b) Fluorescence increased with increasing TAMRA-Pam_2CSK_4 when excited at 525 nm (i.e., the TAMRA excitation wavelength).
